# Hepatitis C cross-genotype immunity and implications for vaccine development

**DOI:** 10.1038/s41598-017-10190-8

**Published:** 2017-09-26

**Authors:** Nazrul Islam, Mel Krajden, Jean Shoveller, Paul Gustafson, Mark Gilbert, Jason Wong, Mark W. Tyndall, Naveed Zafar Janjua, The BC-HTC Team, Amanda Yu, Margot Kuo, Maria Alvarez, Mei Chong, Zahid A. Butt, Nabin Shrestha, Hasina Samji, Seyed Ali Mussavi Rizi

**Affiliations:** 2 British Columbia Centre for Disease Control, Vancouver, BC Canada; 8 Performance Measurement & Reporting, Provincial Health Services Authority, Vancouver, BC Canada; 1 School of Population and Public Health, University of British Columbia, Vancouver, BC Canada; 3 Harvard T.H. Chan School of Public Health, Boston, MA USA; 4 Department of Pathology and Laboratory Medicine, University of British Columbia, Vancouver, BC Canada; 5 British Columbia Centre for Excellence in HIV/AIDS, Vancouver, BC Canada; 6 Department of Statistics, University of British Columbia, Vancouver, BC Canada; 7 Ontario HIV Treatment Network, Toronto, ON Canada

## Abstract

While about a quarter of individuals clear their primary hepatitis C (HCV) infections spontaneously, clearance (spontaneous or treatment-induced) does not confer sterilizing immunity against a future infection. Since successful treatment does not prevent future infections either, an effective vaccine is highly desirable in preventing HCV (re)infection. However, development of an effective vaccine has been complicated by the diversity of HCV genotypes, and complexities in HCV immunological responses. Smaller studies on humans and chimpanzees reported seemingly opposing results regarding cross-neutralizing antibodies. We report a lack of cross-genotype immunity in the largest cohort of people to date. In the adjusted Cox proportional hazards model, reinfection with a heterologous HCV genotype (adjusted Hazard Ratio [aHR]: 0.45, 95% CI: 0.25–0.84) was associated with a 55% lower likelihood of re-clearance. Among those who cleared their first infection spontaneously, the likelihood of re-clearance was 49% lower (aHR: 0.51, 95% CI: 0.27–0.94) when reinfected with a heterologous HCV genotype. These findings indicate that immunity against a particular HCV genotype does not offer expanded immunity to protect against subsequent infections with a different HCV genotype. A prophylactic HCV vaccine boosted with multiple HCV genotype may offer a broader and more effective protection.

## Introduction

Ability to clear an episode of hepatitis C virus (HCV) infection, spontaneously^1,2^ or after a successful treatment, does not protect against a future infection^3–11^. New DAAs are highly effective but prohibitively expensive, and there is no effective vaccine against HCV^12^. As we wait for an effective vaccine against HCV, studies on humans and chimpanzees provide important insights into developing such vaccine^13^. Previous studies in chimpanzees^14–16^, and humans^17,18^, indicated that compared to primary infection, exposure to a subsequent infection was associated a lower peak viremia, overall shortened infection course and lower ALT, and a higher likelihood of viral clearance. While earlier case studies reported similar findings^17,18^, including cross-neutralizing antibodies^17^, this has not been examined in larger population-based studies before. Studies on Chimpanzees reported seemingly opposing results^15,16^. Some animal data reported cross-genotype immunity^15^, while other findings suggested limited protection against heterologous HCV viral strains^16,19^. While these findings are important in understanding the natural history of HCV to lend support to vaccine development, these, and other epidemiological studies^7,17,18,20,21^ had very small sample size that may have compromised their generalizability and their ability to examine factors determining the outcome following HCV reinfection. The studies on humans published so far summarized clearance of reinfection as case reports or case series except one study^22^ that only reported unadjusted estimates, and was unable to examine factors associated with HCV re-clearance adjusting for other potential confounders due to very small sample size (n = 14). In this study, we examined if reinfection with a heterologous HCV genotype had any impact on re-clearance.

## Results

Of 452 cases of reinfection, 357 participants had at least one valid HCV-PCR tests after reinfection. They were followed up for a median of 26.7 (IQR: 12.2–53.8) months post-reinfection. Almost half (49%; n = 175) of them had at least one negative HCV-PCR after reinfection; 121 (34%) of them had two consecutive negative PCR at least 28 days apart (*confirmed* re-clearance), and the rest (15%; n = 54) had either only one negative PCR, or two consecutive negative PCR < 28 days apart (*probable* re-clearance). Individuals included in this study had a median of 12 (IQR: 9–16) HCV-PCR tests overall with a median of 5 (IQR: 3–8; range: 2–33) HCV-PCR tests after reinfection. The median testing interval post-reinfection was 5.5 (IQR: 2.2–12.1) months. Study participants were observed for a median of 24.0 (IQR: 9.2–56.5) months to examine the spontaneous clearance of first infection, and 34.4 (IQR: 16.2–61.8) months to develop reinfection before entering the current study.

Table 1 shows the characteristics of the sample population. The majority of the participants were male (64%; n = 228), and 35–44 years old at reinfection (39%; n = 139). The proportion of people who inject drugs (PWID), and people co-infected with HIV in this sample was 39% (n = 140), and 21% (n = 76), respectively. While more than a fifth (22%; n = 78) had an ongoing history of problematic alcohol use, almost a quarter (24%; n = 86) had a current history of major mental illness. Most of them (90%; n = 323) cleared their first infection spontaneously, and 18% (n = 63) had a reinfection episode with a heterologous HCV genotype.

**Table 1:** Characteristics of participants assessed for hepatitis C virus re-clearance in British Columbia, Canada.

Characteristics	Re-clearance		
	Confirmed* (N = 121)	Probable† (N = 54)	No (N = 182)
Age at HCV reinfection (years)			
<35	37 (30.6)	14 (25.9)	49 (26.9)
35–44	47 (38.8)	25 (46.3)	67 (36.8)
≥45	37 (30.6)	15 (27.8)	66 (36.3)
Birth cohort			
<1964	54 (44.6)	27 (50.0)	81 (44.5)
1965–1974	38 (31.4)	14 (25.9)	61 (33.5)
≥1975	29 (24.0)	13 (24.1)	40 (22.0)
Sex			
Female	39 (32.2)	28 (51.9)	62 (34.1)
Male	82 (67.8)	26 (48.2)	120 (65.9)
Year of HCV diagnosis			
1990–1997	50 (41.3)	15 (27.8)	58 (31.9)
1998–2004	48 (39.7)	25 (46.3)	96 (52.8)
2005–2013	23 (19.0)	14 (25.9)	28 (15.4)
HCV heterologous genotype			
Yes	12 (9.9)	8 (14.8)	43 (23.6)
No	109 (90.1)	46 (85.2)	139 (76.4)
Clearance type^‡^			
Spontaneous clearance	114 (94.2)	50 (92.6)	159 (87.4)
Sustained virological response	7 (5.8)	4 (7.4)	23 (12.6)
HIV**			
Yes	24 (19.8)	8 (14.8)	44 (24.2)
No	97 (80.2)	46 (85.2)	138 (75.8)
Major mental illness***			
Yes	26 (21.5)	14 (25.9)	46 (25.3)
No	95 (78.5)	40 (74.1)	136 (74.7)
Injection drug use***			
Yes	47 (38.8)	24 (44.4)	69 (37.9)
No	74 (61.2)	30 (55.6)	113 (62.1)
Problematic alcohol use***			
Yes	19 (15.7)	16 (29.6)	43 (23.6)
No	102 (84.3)	38 (70.4)	139 (76.4)
Material deprivation quintile at reinfection			
Q1 (most privileged)	16 (13.2)	3 (5.6)	27 (14.8)
Q2	19 (15.7)	11 (20.4)	28 (15.4)
Q3	18 (14.9)	6 (11.1)	24 (13.2)
Q4	32 (26.5)	14 (25.9)	35 (19.2)
Q5 (most deprived)	30 (24.8)	16 (29.6)	62 (34.1)
Unknown	6 (5.0)	4 (7.4)	6 (3.3)
Social deprivation quintile at reinfection			
Q1 (most privileged)	12 (9.9)	4 (7.4)	11 (6.0)
Q2	11 (9.1)	5 (9.3)	20 (11.0)
Q3	14 (11.6)	6 (11.1)	21 (11.5)
Q4	30 (24.8)	11 (20.4)	28 (15.4)
Q5 (most deprived)	48 (39.7)	24 (44.4)	96 (52.8)
Unknown	6 (5.0)	4 (7.4)	6 (3.3)

*Two consecutive negative PCR, at least 28 days apart; ^†^Either one negative PCR, or two consecutive negative PCR but the difference between them was less than 28 days; ^‡^Clearance type of the first HCV infection; **Any time before the last day of follow-up; ***Any time during the study follow-up time. HCV: Hepatitis C Virus; HIV: Human Immunodeficiency Virus; PCR = Polymerase Chain Reaction (RNA testing for HCV).

In the adjusted analysis, reinfection with a heterologous HCV genotype (adjusted Hazard Ratio [aHR]: 0.45, 95% CI: 0.25–0.84), was significantly associated with a 55% lower likelihood of HCV re-clearance (Table 2). Among those who cleared their first infections spontaneously, reinfection with a heterologous genotype was associated with a 49% lower likelihood (aHR: 0.51, 95% CI: 0.27–0.94) of HCV re-clearance adjusting for other potential confounders (Table 3). The propensity score-based IPTW-weighted Cox proportional hazards model also yielded similar results (aHR: 0.32, 95% CI: 0.23–0.45 in the full cohort, and aHR: 0.37, 95% CI: 0.26–0.52 among those who spontaneously cleared their first infection). The results were similar in the sensitivity analysis combining probable and confirmed re-clearance (Supplementary Table 3), and also in the logistic regression models (Supplementary Table 4).

**Table 2:** Unadjusted and adjusted hazard ratios for factors associated with hepatitis C virus re-clearance (confirmed) in British Columbia, Canada.

Characteristics	Unadjusted HR (95% CI)	p-value	Adjusted HR (95% CI)	p-value
Age at HCV reinfection (years)		0.579		0.225
<35	1.27 (0.81–2.01)		1.48 (0.92–2.38)	
35–44	1.11 (0.72–1.70)		1.36 (0.88–2.10)	
≥45	*Ref*		*Ref*	
Birth cohort		0.661		
<1965	0.81 (0.52–1.28)			
1965–1974	0.89 (0.55–1.44)			
≥1975	*Ref*			
Female	0.86 (0.59–1.26)	0.444	0.78 (0.52–1.16)	0.213
Year of HCV diagnosis		0.01		0.02
1990–1997	0.67 (0.41–1.12)		0.69 (0.41–1.16)	
1998–2004	0.47 (0.28–0.77)		0.49 (0.29–0.82)	
2005–2013	*Ref*		*Ref*	
HCV heterologous genotype	0.47 (0.26–0.86)	0.014	0.45 (0.25–0.84)	0.012
Spontaneous clearance‡	1.7 (0.79–3.64)	0.176		
HIV**	0.86 (0.55–1.34)	0.498		
Major mental illness***	0.71 (0.46–1.09)	0.117		
Injection drug use***	0.79 (0.55–1.15)	0.219		
Problematic alcohol use***	0.50 (0.30 –0.81)	0.005	0.47 (0.29–0.78)	0.004
Material deprivation quintile at reinfection		0.803		
Q1 (most privileged)	*Ref*			
Q2	0.95 (0.48–1.86)			
Q3	1.03 (0.52–2.04)			
Q4	1.03 (0.56–1.88)			
Q5 (most deprived)	0.74 (0.40–1.36)			
Unknown	1.05 (0.41–2.69)			
Social deprivation quintile at reinfection		0.344		
Q1 (most privileged)	*Ref*			
Q2	0.68 (0.30–1.55)			
Q3	0.83 (0.38–1.80)			
Q4	0.98 (0.50 –1.92)			
Q5 (most deprived)	0.61 (0.32–1.14)			
Unknown	0.85 (0.32–2.26)			

^‡^Clearance type of the first HCV infection. **Used as a time-varying covariate. ***Any time during the study follow-up time. HCV: Hepatitis C Virus; HIV: Human Immunodeficiency Virus; HR: Hazard Ratio: CI: Confidence Interval.

**Table 3:** Unadjusted and adjusted hazard ratios for factors associated with hepatitis C virus re-clearance (confirmed) in British Columbia, Canada (restricted to those who spontaneously cleared their first HCV infection).

Characteristics	Unadjusted HR (95% CI)	p-value	Adjusted HR (95% CI)	p-value
Age at HCV reinfection (years)		0.541		0.211
<35	1.30 (0.81–2.10)		1.50 (0.92–2.44)	
35–44	1.19 (0.75–1.87)		1.42 (0.90–2.24)	
≥45	*Ref*		*Ref*	
Birth cohort		0.47		
<1965	0.78 (0.49–1.24)			
1965–1974	0.96 (0.59–1.57)			
≥1975	*Ref*			
Female	0.83 (0.56–1.22)	0.337	0.75 (0.49–1.12)	0.16
Year of HCV diagnosis		0.019		0.024
1990–1997	0.63 (0.38–1.06)		0.63 (0.37–1.07)	
1998–2004	0.48 (0.29–0.80)		0.49 (0.29–0.82)	
2005–2013	*Ref*		*Ref*	
HCV heterologous genotype	0.57 (0.32–1.04)	0.069	0.51 (0.27–0.94)	0.031
HIV**	0.91 (0.58–1.42)	0.672		
Major mental illness***	0.79 (0.50–1.23)	0.287		
Injection drug use***	0.87 (0.60–1.26)	0.459		
Problematic alcohol use***	0.53 (0.33–0.87)	0.012	0.50 (0.30–0.83)	0.008
Material deprivation quintile at reinfection		0.868		
Q1 (most privileged)	*Ref*			
Q2	0.92 (0.46–1.86)			
Q3	1.00 (0.49–2.01)			
Q4	1.01 (0.54–1.88)			
Q5 (most deprived)	0.74 (0.39–1.39)			
Unknown	0.98 (0.38–2.54)			
Social deprivation quintile at reinfection		0.25		
Q1 (most privileged)	*Ref*			
Q2	0.78 (0.34–1.80)			
Q3	0.71 (0.30–1.66)			
Q4	1.22 (0.61–2.44)			
Q5 (most deprived)	0.69 (0.36–1.33)			
Unknown	0.90 (0.33–2.43)			

**Used as a time-varying covariate. ***Any time within the study follow-up time. HCV: Hepatitis C Virus; HIV: Human Immunodeficiency Virus; HR: Hazard Ratio: CI: Confidence Interval.

We further analyzed the data to see if re-clearance was associated with the sequence of HCV genotypes (GT). Compared to those who were infected with GT1 in both of their episodes, those infected with GT2/3 in both their infection episodes showed a slightly reduced likelihood of re-clearance but it did not reach statistical significance (aHR: 0.56, 95% CI: 0.21–1.51, p = 0.254). Further, compared to those without reinfection with a heterologous HCV genotype, those who were infected with GT1 followed by a second infection with GT2/3 showed a higher likelihood of re-clearance (aHR: 1.31, 95% CI: 0.47–3.65, p = 0.601), but those who were infected with GT2/3 followed by a second infection with GT1 showed a lower likelihood of re-clearance (aHR: 0.62, 95% CI: 0.23–1.70, p = 0.354). Again, none of these reached statistical significance.

## Discussion

To our knowledge, this is the largest study thus far to examine HCV re-clearance in humans. Consistent with earlier reports, the rate of spontaneous clearance of reinfection was much higher than that of first infection^2^. We also showed that reinfection with a heterologous HCV genotype was associated with a significant reduction in the likelihood of re-clearance among those who cleared their first HCV episode spontaneously indicating a lack of cross-genotype protective immune response against subsequent infections.

In our study, the proportion of confirmed re-clearance was 34%. Studies previously reported a wide range of estimates (0–100%)^17,18,20,22^. Even though the rate of re-clearance (per person-year) was not reported earlier, the proportion of re-clearance was reported to be higher than the clearance of the first infection in smaller studies in chimpanzees and humans^17,18,20,22,23^. Our findings align with these findings.

We found a significantly reduced likelihood of HCV re-clearance when reinfected with a heterologous genotype, and among those who had an ongoing history of problematic alcohol use. Though not evaluated in the context of HCV re-clearance, alcohol abuse was reported to be associated with reduced odds of spontaneous clearance of HCV^24^. Alcohol abuse was found to worsen the clinical outcomes of HCV in humans by altering the immune response to HCV, particularly by inhibiting the T-cell activating capacity^25,26^. The only study that examined the potential factors associated with re-clearance found females with the IFNL4 rs12979860 CC genotype a significant predictor, even though it was not adjusted for other potential confounders^22^. While female sex was not a significant predictor in our adjusted analysis, we did not have data on IFNL4, and so we were not able to examine this. Further studies with sufficient sample size to adjust for potential confounders are required to validate these findings.

Among those who cleared their previous HCV episode spontaneously, we found that reinfection with a heterologous genotype was associated with a significantly lower likelihood of re-clearance. While earlier case studies reported similar findings^17,18^, including cross-neutralizing antibodies^17^, this has not been examined in larger population-based studies before. Studies on chimpanzees reported seemingly opposing results^15,16^. Some animal data reported cross-genotype immunity^15^, while other findings suggested limited protection against heterologous HCV viral strains^16,19^.

Besides testing the robustness of our findings by a series of additional analysis including the application of propensity score methods to address the issue of confounding by indication, we further assessed the effect of sequence of genotypes on re-clearance and found that people who were infected with genotype 1 followed by genotype 2/3 were more likely to clear their second infection spontaneously while those infected with genotype 2/3 in their first infection followed by genotype 1 infection were less likely to clear their 2^nd^ infection. None of these associations reached statistical significance. Because this was not in our primary hypotheses tested in this study, caution should be exercised in interpreting these findings, and we recommend further studies with larger sample size to characterize impact of specific genotype sequences.

To our knowledge, this is the first population-based comprehensive study with the largest sample size to examine spontaneous clearance of HCV reinfection. We collected data on other potential confounders to examine the factors associated with HCV re-clearance making it the first study to do so. However, some of the limitations of this study include our inability to examine the impact of ethnicity, and genetic markers including IFNL4.

## Conclusions

While we wait for an effective vaccine to offer sterilizing immunity, it is probably more pragmatic to develop a prophylactic vaccine that augments spontaneous clearance of HCV inducing partial protective immunity^12^. Lessons from other vaccines (e.g. those against hepatitis B, human papilloma, influenza, and varicella zoster)^27^, which, instead of providing sterilizing immunity, protect against persistent infections and result in a weakened course of infections may be relevant for HCV. Within the context of limited protection against heterologous genotypes shown in our study, immunization with prophylactic vaccine, and boosting with different HCV genotypes have been suggested for a broader and more effective protection^19^.

## Methods

The data for this analysis is based on the BC Hepatitis Testers Cohort (BC-HTC)^2,28,29^ that includes all individuals tested for HCV or HIV at the BC Centre for Disease Control Public Health Laboratory (BCCDC-PHL) or reported case of HCV, HBV, or HIV, and linked with data on demographic characteristics^S6^, medical visits^S2^, hospitalizations^S1^, and prescription drugs (including HCV treatment)^S3,S4^ (Supplementary Table 1). BCCDC-PHL is the centralized laboratory for most serology (>95%) and all HCV RNA (PCR) testing in BC, and thus provides a unique tool to monitor and assess the impact of HCV treatment and harm reduction initiatives across the natural history of HCV. Details of the BC-HTC including creation, linkage, characteristics, and matching have been reported previously^28^. Current analysis included all HCV reinfected individuals who had at least one valid HCV-PCR after reinfection, and who did not receive HCV treatment after reinfection.

An HCV case is an individual who tested positive for either HCV antibody or HCV-PCR or genotype, or who was reported as a case of HCV in the Integrated Public Health Information System (iPHIS)^28^. HCV diagnosis date was the date of HCV positive test for those testing positive on the first test while it was the midpoint between the last negative HCV date and the first time they tested positive for HCV for those who tested negative for HCV at first test, and subsequently tested positive for HCV (seroconverters).

Spontaneous clearance was defined as two consecutive negative HCV-PCR tests, at least 28 days apart^22^, following HCV diagnosis without treatment. The date of spontaneous clearance was calculated as the midpoint between the last positive and first negative PCR following HCV diagnosis^22^. Sustained virological response (SVR) was defined as two consecutive negative HCV-PCR tests, at least 28 days apart^22^, at ≥12 weeks post-treatment completion (the date of last dispensation of HCV treatment plus the days the drug was dispensed for)^30^. The date of SVR was calculated as the midpoint between the 12-weeks post-treatment end date and the subsequent first negative PCR date.

HCV reinfection was defined as a positive HCV-PCR following clearance (spontaneous clearance or SVR) of first infection. The date of reinfection was calculated as the midpoint between the last negative PCR and the first positive PCR following clearance of the first infection.

HCV re-clearance (*confirmed*) was defined as two consecutive negative PCR tests, at least 28 days apart, after reinfection. Those who had only one negative HCV-PCR, or two consecutive negative PCR within a 28-day time frame, were defined as *probable* re-clearance. The date of re-clearance was the midpoint between the last positive and the first negative PCR after reinfection. Time from reinfection to re-clearance was the main outcome variable.

HIV cases were identified based on reporting to the HIV/AIDS Information System (HAISYS), which includes all HIV/AIDS cases reported in BC, or HIV lab tests as per provincial guidelines^31^. Additional cases who tested without nominal information were identified through a previously validated algorithm based on medical visits and/or hospital admissions described elsewhere^28^. Major mental illness, injection drug use (IDU), and problematic alcohol use were defined based on diagnostic codes from medical services plan or discharge abstract database (DAD)﻿. Details including the International Classification of Diseases [ICD]) codes have been provided in Supplementary Table 2. The heterologous genotype was defined as reinfections with an HCV genotype other than the one in first infection. We also assessed the social and material deprivation based on Québec Index of Material and Social Deprivation.

Variables based on a-priori hypotheses, and those significant at 0.10 level in the unadjusted analysis were included in the multivariable Cox proportional hazards (PH) models. For the main analyses, time to *confirmed* re-clearance was the main outcome variable (probable and no re-clearance were censored). The last day of follow-up was the date of re-clearance, or last positive date after reinfection or the date a person received any treatment, whichever came first. Age at reinfection, sex, and year of HCV diagnosis were included in all the adjusted analyses irrespective of their statistical significance in the unadjusted analysis. HIV was used as a time-varying covariate in all the analyses.

To assess the role of reinfection with a heterologous genotype on spontaneous re-clearance among those who spontaneously cleared their first infection, we performed an additional analysis restricted to those who cleared their first infection spontaneously. Multinomial logistic regression was fit to assess the robustness of the time-to-event analysis. Propensity score methods were applied to calculate the inverse probability of treatment weight (IPTW). IPTW-weighted Cox PH model was fitted to address the issue of treatment-indication bias^5^.

In the sensitivity analysis, the *confirmed* and *probable* re-clearance groups were merged as re-clearance. Logistic regression was fit to assess the robustness of this analysis. All the analyses were conducted in [SAS/STAT] Software version [9.4]. All the tests were two-sided at a significance level of 0.05.

### Ethics and consent to participate

Data linkage to establish the BC-HTC was performed under the BCCDC’s public health mandate. The Behavioral Research Ethics Board at the University of British Columbia reviewed and approved the study (H14-01649). All experiments were performed in accordance with the relevant guidelines and regulations at BCCDC. Patient consent was not required as study used de-identified linked administrative healthcare datasets. No identifying information of the study participants has been included in this study.

### Data availability

Data are available from the BC Centre for Disease Control Institutional Data Access for researchers who meet the criteria for access to confidential data. Requests for the data may be sent to Dr. Naveed Janjua (naveed.janjua@bccdc.ca).

### Disclaimer

All inferences, opinions, and conclusions drawn in this publication are those of the author(s), and do not necessarily reflect the opinions or policies of the [British Columbia] Ministry of Health.

## Electronic supplementary material

Supplementary Tables
