# Supplementary material for: Hepatitis C cross-genotype immunity and implications for vaccine development
Source: Sci Rep. 2017 Sep 26;7:12326. doi: 10.1038/s41598-017-10190-8 (PMC5615075; doi:10.1038/s41598-017-10190-8)
Supplement: Supplementary file 1 — Supplementary Tables [file 41598_2017_10190_MOESM1_ESM.doc]

**Hepatitis C cross-genotype immunity and implications for vaccine development**

Nazrul Islam, PhD^1,2,3^, Mel Krajden, MD^2,4^, Jean Shoveller, PhD^1,5^, Paul Gustafson, PhD^6^, Mark Gilbert, MD^2,7^, Jason Wong, MD^1,2^, Mark W Tyndall, ScD^1,2^, Naveed Zafar Janjua, DrPH^1,2^, BC-HTC Team^*^

1. School of Population and Public Health, University of British Columbia, Vancouver, BC, Canada.
2. British Columbia Centre for Disease Control, Vancouver, BC, Canada.
3. Harvard T.H. Chan School of Public Health, Boston, MA, USA.
4. Department of Pathology and Laboratory Medicine, University of British Columbia, Vancouver, BC, Canada.
5. British Columbia Centre for Excellence in HIV/AIDS, Vancouver, BC, Canada.
6. Department of Statistics, University of British Columbia, Vancouver, BC, Canada.
7. Ontario HIV Treatment Network, Toronto, ON, Canada.

**Supplementary Table 1: Criteria and Data Sources for the BC Hepatitis Testers Cohort (BC-HTC)**

| **Criteria for Inclusion in BC-HTC** | |
| --- | --- |
| All individuals: | |
| - tested at the centralized provincial laboratory for HCV or HIV OR | |
| - reported by BC public health as a confirmed case of HCV OR | |
| - reported in BC enhanced surveillance system as a confirmed case of HIV or AIDS (all reports) OR | |
| - reported by BC public health as a confirmed case of HBV OR | |
| - included in BC Enhanced Strain Surveillance System (EHSSS) as an acute HBV or HCV case | |
| - All individuals meeting at least one the above criteria were linked internally across all their tests and case reports. Those with a valid personal health number (PHN) were then sent for deterministic linkage with province-wide Cancer and Ministry of Health (MoH) datasets | |
| **Provincial Communicable Disease Data Sources:** | **Data Date Ranges:** |
| BC-PHMRL HIV laboratory testing datasets (tests: ELISA, Western blot, NAAT, p24, culture) | 1988–2013 |
| BC-PHMRL HCV laboratory tests datasets (tests: antibody, HCV RNA, genotyping) | 1992–2013 |
| HIV/AIDS Information System (HAISYS) (public health HIV/AIDS case reports) | 1980–2013 |
| Integrated Public Health information System (iPHIS) (public health case reports of HCV, HBV, and TB) | 1990–2013 |
| Enhanced Strain Surveillance System (EHSSS) (risk factor data on a subset of acute HCV and acute HBV cases) | 2000–2013 |
| Cancer and MoH Administrative Data Sources: | Data Date Ranges: |
| BC Cancer Registry (BCCR) (primary tumour registry, excludes metastatic cancers) | 1970–2012 |
| Discharge Abstracts Dataset (DAD) (hospitalization records)^S1^ | 1985–2013Q1 |
| Medical Services Plan (MSP) (physician diagnostic and billing data)^S2^ | 1990–2012 |
| PharmaCare/PharmaNet (Pharma) (prescription drug dispensations)^S3, S4^ | 1985–2012 |
| BC Vital Statistics (VS) (deaths registry)^S5^ | 1985–2013 |
| The final BC-HTC comprises all individuals successfully linked on PHN to the MoH Client Roster^S6^ (a registry of all BC residents enrolled in the publicly-funded universal healthcare system) | |

HCV: Hepatitis C Virus; HBV: Hepatitis B Virus; HIV/AIDS: Human Immunodeficiency Virus/Acquired Immunodeficiency Syndrome; BC-PHMRL: BC Public Health Microbiology and Reference Laboratory: RNA: Ribonucleic Acid; PCR: Polymerase Chain Reaction.

**Supplementary References**:

1. British Columbia Ministry of Health [creator]. Discharge Abstract Database (Hospital Separations). British Columbia Ministry of Health [publisher]. Data Extract. MOH (2013). 2014. <http://www.health.gov.bc.ca/data/>
2. British Columbia Ministry of Health [creator]. Medical Services Plan (MSP) Payment Information File. British Columbia Ministry of Health [publisher]. Data Extract. MOH (2013). 2014. <http://www.health.gov.bc.ca/data/>
3. British Columbia Ministry of Health [creator]. PharmaCare. British Columbia Ministry of Health [publisher]. Data Extract. MOH (2013). 2014. <http://www.health.gov.bc.ca/data/>
4. British Columbia Ministry of Health [creator]. PharmaNet. British Columbia Ministry of Health [publisher]. Data Extract. MOH (2013). 2014. <http://www.health.gov.bc.ca/data/>
5. BC Vital Statistics Agency [creator]. Vital Statistics Deaths. BC Vital Statistics Agency [publisher]. Data Extract. BC Vital Statistics Agency (2014). 2014.
6. British Columbia Ministry of Health [creator]. Client Roster (Client Registry System/Enterprise Master Patient Index). British Columbia Ministry of Health [publisher]. Data Extract. MOH (2013). 2014. <http://www.health.gov.bc.ca/data/>

**Supplementary Table 2: Definitions for comorbid conditions for the BC Hepatitis Testers Cohort (BC-HTC) and current analysis**

| **Major Mental Illness**  Major mental illness was flagged at the first occurrence of a hospitalization diagnostic code OR 2 MSP diagnostic codes from a psychiatrist visit for schizophrenic, bipolar, delusional, nonorganic psychotic, adjustment, anxiety, dissociative, personality and major depressive disorders.  Physician Billing Data: MSP ICD-9 diagnostic codes: starting with 295-298, 300-301, 308-309, 311 AND claim specialty = 3  Hospitalization Data: DAD1/ICD-9-CM: starting with 295-298, 300-301, 308-309, 311; DAD2/ICD-10-CA: starting with F20-F25, F28-F34, F38-F45, F48, F60-F61 |
| --- |
| **Injection Drug Use**  Illicit Drug Use was defined at the first occurrence of 1 MSP or 1 hospitalization diagnostic code for major drug-related diagnoses involving addiction, dependence, and drug-induced mental disorders; illicit drug use most likely to be injectables (e.g. excluding cannabis), or illicit use of prescribed drugs including: hallucinogens, barbituates/tranquillizers, sedatives, hypnotics, anxiolytics, opioids, cocaine, amphetamine, volatile solvents; or discharge to drug rehabilitation, counselling, and surveillance.  Physician Billing Data: MSP ICD-9 diagnostic codes: starting with 292, 3040, 3042, 3044, 3046-9, 3054-7, 3059, 6483, 9650, 9697, 970, E8500, or exact codes V6542 or fee item = 39  Hospitalization Data: DAD1/ICD-9-CM: starting with 292, 3040, 3042, 3044, 3046-9, 3054-7, 3059, 6483, 9650, 9697, 970, E8500; DAD2/ICD-10-CA: starting with F11, F13-5, F18, F19, T42, or exact codes T401, T402, T404-6, T436, T438, T439, T507. |
| **Problematic Alcohol Use**  Problematic alcohol use was defined at the first occurrence of 2 MSP or 1 hospitalization codes for major alcohol-related diagnoses including alcoholic mental disorders and dependence/abuse syndromes; alcoholic polyneuropathy, myopathy, cardiomyopathy; pseudo Cushing’s syndrome; or discharge to alcohol rehabilitation, counselling, or surveillance.  Physician Billing Data: MSP ICD-9 diagnostic codes: starting with 291, 303, 3050, 3575, 4255  Hospitalization Data: DAD1/ICD-9-CM: starting with 291, 303, 3050,3575, 4255; DAD2/ICD-10-CA: starting with F10, E244, G312, G621, G721, I426, Z502, Z714 |

Supplementary Table 3: Unadjusted and adjusted hazard ratios for factors associated with HCV re-clearance (probable + confirmed) in British Columbia, Canada

| **Characteristics** | **Unadjusted HR**  **(95% CI)** | **p-value** | **Adjusted HR**  **(95% CI)** | **p-value** |
| --- | --- | --- | --- | --- |
| Age at HCV reinfection (year) |  | 0.429 |  |  |
| < 35 | 1.27 (0.86-1.87) |  |  |  |
| 35-44 | 1.21 (0.85-1.73) |  |  |  |
| ≥ 45 | *Ref* |  |  |  |
| Birth Cohort |  | 0.537 |  | 0.555 |
| < 1965 | 0.81 (0.56-1.19) |  | 0.90 (0.60-1.34) |  |
| 1965-1974 | 0.83 (0.55-1.25) |  | 1.10 (0.72-1.69) |  |
| ≥ 1975 | *Ref* |  | *Ref* |  |
| Female | 1.13 (0.83-1.53) | 0.44 | 1.03 (0.74-1.42) | 0.877 |
| Year of HCV diagnosis |  | 0.0001 |  | 0.001 |
| 1990-1997 | 0.51 (0.34-0.77) |  | 0.52 (0.34-0.81) |  |
| 1998-2004 | 0.43 (0.28-0.64) |  | 0.45 90.30-0.68) |  |
| 2005-2013 | *Ref* |  | *Ref* |  |
| HCV heterologous genotype | 0.56 (0.35-0.89) | 0.014 | 0.57 (0.35-0.93) | 0.024 |
| Spontaneous clearance‡ | 1.54 (0.84-2.84) | 0.176 |  |  |
| HIV** | 0.77 (0.53-1.13) | 0.185 |  |  |
| Major mental illness*** | 0.77 (0.54-1.09) | 0.138 |  |  |
| Injection drug use*** | 0.85 (0.63-1.15) | 0.295 |  |  |
| Problematic alcohol use*** | 0.66 (0.45-0.95) | 0.027 | 0.61 (0.42-0.89) | 0.011 |
| Material deprivation quintile at reinfection |  | 0.689 |  |  |
| Q1 (most privileged) | *Ref* |  |  |  |
| Q2 | 1.25 (0.7-2.23) |  |  |  |
| Q3 | 1.15 (0.63-2.12) |  |  |  |
| Q4 | 1.24 (0.72-2.13) |  |  |  |
| Q5 (most deprived) | 0.95 (0.56-1.63) |  |  |  |
| Unknown | 1.48 (0.69-3.2) |  |  |  |
| Social deprivation quintile at reinfection |  | 0.332 |  |  |
| Q1 (most privileged) | *Ref* |  |  |  |
| Q2 | 0.77 (0.39-1.55) |  |  |  |
| Q3 | 0.91 (0.47-1.76) |  |  |  |
| Q4 | 1.03 (0.58-1.84) |  |  |  |
| Q5 (most deprived) | 0.69 (0.4-1.19) |  |  |  |
| Unknown | 1.09 (0.5-2.41) |  |  |  |

‡ Clearance type of the first HCV infection (ref.: sustained virological response); ** used as a time-varying covariate; *** Any time during the study follow-up time; HCV: Hepatitis C Virus; HIV: Human Immunodeficiency Virus; HR: Hazard Ratio: CI: Confidence Interval.

Supplementary Table 4: Adjusted odds ratios from logistic regression examining factors associated with HCV re-clearance in British Columbia, Canada

| **Characteristics** | **Probable† re-clearance** | **Confirmed* re-clearance** | **Confirmed* + Probable† re-clearance** |
| --- | --- | --- | --- |
| Age at HCV reinfection (year) |  |  |  |
| < 35 | 0.95 (0.40-2.26) | 1.66 (0.88-3.12) | 1.39 (0.79-2.46) |
| 35-44 | 1.66 (0.78-3.54) | 1.54 (0.87-2.74) | 1.60 (0.95-2.67) |
| ≥ 45 | *Ref* | *Ref* | *Ref* |
| Female | 1.80 (0.92-3.52) | 0.75 (0.44-1.27) | 1.00 (0.63-1.60) |
| Year of HCV diagnosis |  |  |  |
| 1990-1997 | 0.50 (0.20-1.23) | 1.11 (0.55-2.25) | 0.86 (0.46-1.63) |
| 1998-2004 | 0.52 (0.23-1.17) | 0.68 (0.34-1.34) | 0.62 (0.34-1.13) |
| 2005-2013 | *Ref* | *Ref* | *Ref* |
| HCV Heterologous genotype | 0.71 (0.29-1.72) | 0.34 (0.16-0.69) | 0.43 (0.23-0.79) |
| Problematic Alcohol Use^‡^ | 1.18 (0.58-2.41) | 0.55 (0.29-1.01) | 0.72 (0.43-1.23) |

No clearance was the reference group.

* Two consecutive negative PCR, at least 28 days apart; † Either one negative PCR, or two consecutive negative PCR but the difference between them was less than 28 days; ‡ Any time within the study follow-up time.

HCV: Hepatitis C Virus; HIV: Human Immunodeficiency Virus; OR: Odds Ratio: CI: Confidence Interval
